# The complete mitochondrial genome sequences of potato (*Solanum tuberosum* L., Solanaceae)

**DOI:** 10.1080/23802359.2017.1398607

**Published:** 2017-11-07

**Authors:** Kwang-Soo Cho, Ji-Hong Cho, Ju-Sung Im, Jang-Gyu Choi, Young-Eun Park, Su-Young Hong, Min Kwon, Jin-Ho Kang, Tae-Ho Park

**Affiliations:** a Highland Agriculture Research Institute, Natioanl institute of Crop Science, Rural Development Administration, Pyeongchang, South Korea;; b Graduate School of International Agricultural Technology and Crop Biotechnology Institute, Seoul National University, Pyeongchang, South Korea;; c Department of Horticulture, Daegu University, Gyeongsan, South Korea

**Keywords:** Potato, *Solanum tuberosum*, Mitochondrial genome, Solanaceae

## Abstract

Potato (*Solanum tuberosum*) from the Solanaceae is the fourth most important food crop worldwide. In this study, five complete mitochondrial genome sequences of *S. tuberosum* were characterized through *de novo* assembly of whole genome sequencing data. The resulting circular mitochondrial DNA molecules ranged from 49,171 bp to 297,014 bp in size and contained a total of 80 non-redundant genes, comprising 34 protein-coding genes, 24 hypothetical open reading frames, 19 tRNA genes, and 3 rRNA genes. Phylogenetic analysis using common protein-coding sequences confirmed that *S. tuberosum* belongs to the Solanoideae subfamily in the Solanaceae family.

Mitochondrial (mt) genomes of higher plants are more complex and encode a higher number of genes compared to those of other organisms. Their distinct features are the frequent uptake of foreign DNA by gene transfer (Richardson and Palmer 2007; Goremykin et al. 2009), very low mutation rate (Wolfe et al. 1987), and dynamic structure (Palmer and Herbon 1988) with an unusual size variation (Alverson et al. 2010). Furthermore, they play important roles in plant development and productivity (Ogihara et al. 2005; Li et al. 2009). Nevertheless, complete sequences of mt genomes from higher plants are rarely acquired, and none has been reported for *Solanum* species since the mt genome of *Nicotiana tabacum*, another member of the Solanaceae family, was reported (Sugiyama et al. 2005). In this study, we report the complete mt genome sequences of *Solanum tuberosum* L. (potato), one of the most important crops worldwide.

Total genomic DNA was extracted from fresh leaves of *S. tuberosum* (breeding line no. PT56 maintained in vitro plant in HARI) grown in Highland Agriculture Research Institute (HARI), National Institute of Crop Science, Rural Development Administration, Pyeongchang, South Korea, and used to construct a paired-end (PE) library with ∼670 bp insert size according to standard Illumina PE library protocol. The library was sequenced using an Illumina MiSeq platform at LabGenomics (Seongnam, Korea). High quality PE reads of about 4.1 Gb were employed for *de novo* assembly using CLC genome assembler (v. beta 4.6; Qiagen, Aarhus, Denmark) as described previously (Cho et al. 2015). Contigs with mitochondrial genome sequences were selected from initial assembled contigs, of which the longest mitochondrial contig was set as the initiation seed contig. The contigs were extended and joined by a series of PE read mappings and gap-fillings to generate final complete mitochondrial genome sequences. The assembled sequences were manually inspected based on read mapping status and BLASTN searches. The genome sequence was annotated using GeSeq (Tillich et al. 2017) and manual curation based on BLAST searches.

Five mitochondrial genome sequences of *S. tuberosum* were completed. The circular DNA molecules were 49,171 bp, 49,230 bp, 112,800 bp, 247,843 bp, and 297,014 bp long (GenBank accession nos. MF989953–MF989957). The largest mitochondrial genome sequence (297,014 bp) included sequences identical to the 247,843-bp and 49,171-bp mitochondrial genomes. A total of 80 non-redundant genes were annotated in the five genomes, comprising 34 protein-coding gene, 24 hypothetical open reading frames, 19 tRNA genes, and three rRNA genes. Among protein-coding genes, exons of trans-splicing *nad2* were dispersed on different genomes. Exons 1 and 2 were present only on the 49,230-bp genome, while Exons 3, 4, and 5 were found in two copies on 297,014-bp and 247,843-bp genomes.

Phylogenetic analysis based on multiple alignments of common 23 protein-coding sequences in the mt genomes confirmed that *S. tuberosum* belongs to the Solanoideae subfamily in the Solanaceae family (Figure 1).

**Figure 1.**
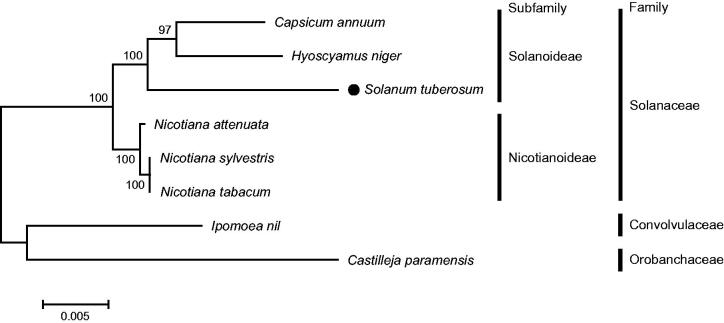
Maximum-likelihood phylogenetic tree of *Solanum tuberosum* and related taxa based on mitochondrial genome sequences. Common protein-coding sequences in mitochondrial genome sequences were aligned using MAFFT (http://mafft.cbrc.jp/alignment/server/index.html) and used to generate maximum-likelihood phylogenetic tree by MEGA 6.0 (Tamura et al. 2013). Numbers on the nodes indicate bootstrap support values (>50%) from 1000 replicates. Scale bar represents the number of nucleotide substitution per site. Mitochondrial genome sequences used for this tree are *Capsicum annuum*, NC_024624; *Castilleja paramensis*, NC_031806; *Hyoscyamus niger*, NC_026515; *Ipomoea nil*, NC_031158; *Nicotiana attenuata*, MF579563; *N. sylvestris*, NC_029805; *N. tabacum,* NC_006581; *Solanum tuberosum,* MF989953–MF989957.
